# PW01-038 – Genomewide association study of Still’s disease

**DOI:** 10.1186/1546-0096-11-S1-A91

**Published:** 2013-11-08

**Authors:** MJ Ombrello, EF Remmers, E Zeggini, W Thomson, DL Kastner, P Woo

**Affiliations:** 1 Translational Genetics and Genomics Unit, NIAMS, Bethesda, MD, USA; 2 Inflammatory Disease Section, NHGRI, Bethesda, MD, USA; 3 Applied Statistical Genetics Group, Wellcome Trust Sanger Institute, Hinxton, UK; 4 Arthritis Research UK Epidemiology Unit, University of Manchester, Manchester, UK; 5 Center of Paediatric and Adolescent Rheumatology, University College London, London, UK

## Introduction

Still’s disease or systemic juvenile idiopathic arthritis (sJIA) is a severe inflammatory disease of childhood characterized by periods of daily spiking fever, evanescent skin rash, severe arthritis, serositis, lymphoid hyperplasia, and, in up to half of cases, macrophage activation syndrome. Although thought to have a genetic component, the causes of sJIA are unknown.

## Objectives

To identify genetic factors that contribute to sJIA susceptibility.

## Methods

We generated single nucleotide polymorphism (SNP) genotypes from the genomic DNA of 988 children with sJIA and 514 healthy control subjects. These data were combined with SNP genotypes *in silico* from 7370 additional healthy control subjects. After dividing the dataset into 9 strata by country of origin, we excluded samples and markers that did not meet our strict quality requirements. We performed haplotype phasing with ShapeIT, SNP imputation with IMPUTE2, and association testing with SNPTEST independently in each stratum. The results of association testing were subjected to fixed- and random-effects meta-analyses with GWAMA. A second round of more intensive “deep imputation” was performed in each region with p_meta_<1E-7. Using the directly genotyped SNP data, we used imputation to deduce classical HLA types in each stratum. Significant associations were further evaluated with multivariate logistic regression using SNPTEST and SNP & Variation Suite 7.

## Results

Using the above method, we ultimately tested a panel of over 1.6M SNPs for association with sJIA. Using meta-analysis of SNP association data from 9 strata, we identified 2 sJIA-associated regions that exceeded the stringent threshold for genome wide significance (p_meta_<5E-8). The strongest association was located in the major histocompatibility complex locus, with one SNP nearest to *HLA-DRB1* (**rs112638393**: p_meta_=1.6E-10, OR 1.5 [1.3, 1.7]) and a second located nearest to *BTNL2* (**rs115945836**: p_meta_=2.4E-10, OR 2.8 [2.0, 3.9]). Conditioning on the effect of rs112638393 accounted for the majority of the effect around *HLA-DRB1*, while revealing a significant, independent association signal spanning *BTNL2* and *HLA-DRA*. Additionally, meta-analysis of the imputed HLA type associations from 8 strata revealed a strong association between HLA-DRB1*1101 and sJIA (p_meta_=1.2E-8, OR 2.1 [1.6, 2.7]). The second strongest regional association, which also exceeded genome wide significance, was located on Chr 1 nearest to *LOC284661* (**rs16838915:** p_meta_=5.4E-9, OR 2.0 [1.6, 2.5]). Logistic regression analysis demonstrated no residual association signal in this region after conditioning on rs16838915. In total, our study identified 11 loci that were suggestive of association with sJIA (p<5E-5).

## Conclusion

We have performed a genome-wide association study of a large collection of sJIA patients. We have identified 2 sJIA susceptibility loci, *HLA-DRB1* and *LOC284661*, both of which have large effect sizes. The association of HLA-DRB1*1101 with sJIA suggests that antigen presentation and the adaptive immune system are involved in sJIA, an idea that would be further supported by involvement of either *HLA-DRA* or *BTNL2*. The specific roles of each of these loci in the pathogenesis of sJIA remain to be elucidated.

## Disclosure of interest

None declared

